# Effects of proximal priority and distal priority robotic priming techniques with impairment-oriented training of upper limb functions in patients with chronic stroke: study protocol for a single-blind, randomized controlled trial

**DOI:** 10.1186/s13063-021-05561-6

**Published:** 2021-09-08

**Authors:** Yi-chen Lee, Yi-chun Li, Keh-chung Lin, Chia-ling Chen, Yi-hsuan Wu, Chihchieh Kuo, Yi-ping Yeh, Ting-xuan Liu

**Affiliations:** 1 School of Occupational Therapy, College of Medicine, National Taiwan University, No.17, F4, Xu Zhou Road, Zhongzheng Dist Taipei City, 100 Taiwan; 2 Division of Occupational Therapy, Department of Physical Medicine and Rehabilitation, National Taiwan University Hospital, No.1, Changde St., Zhongzheng Dist Taipei City, 100 Taiwan; 3 Graduate Institute of Early Intervention, Chang Gung University, No.259, Wenhua 1st Rd., Guishan Dist., Taoyuan City, 33302 Taiwan; 4 Department of Physical Medicine and Rehabilitation, Linkou Chang Gung Memorial Hospital, No. 5, Fuxing St., Guishan Dist., Taoyuan City, 33305 Taiwan; 5 Rehabilitation Department, Feng Yuan Hospital, Ministry of Health and Welfare, No.100, An-Kan Road, Fengyuan Dist Taichung City, 420 Taiwan

**Keywords:** Stroke, Upper extremity rehabilitation, Proximal priority, Distal priority, Robotic therapy, Bilateral motor priming, Impairment-oriented training, Randomized controlled trial

## Abstract

**Background:**

The sequence of establishing a proximal stability or function before facilitation of the distal body part has long been recognized in stroke rehabilitation practice but lacks scientific evidence. This study plans to examine the effects of proximal priority robotic priming and impairment-oriented training (PRI) and distal priority robotic priming and impairment-oriented training (DRI).

**Methods:**

This single-blind, randomized, comparative efficacy study will involve 40 participants with chronic stroke. Participants will be randomized into the PRI or DRI groups and receive 18 intervention sessions (90 min/day, 3 days/weeks for 6 weeks). The Fugl-Meyer Assessment Upper Extremity subscale, Medical Research Council Scale, Revised Nottingham Sensory Assessment, and Wolf Motor Function Test will be administered at baseline, after treatment, and at the 3-month follow-up. Two-way repeated-measures analysis of variance and the chi-square automatic interaction detector method will be used to examine the comparative efficacy and predictors of outcome, respectively, after PRI and DRI.

**Discussion:**

Through manipulating the sequence of applying wrist and forearm robots in therapy, this study will attempt to examine empirically the priming effect of proximal or distal priority robotic therapy in upper extremity impairment-oriented training for people with stroke. The findings will provide directions for further studies and empirical implications for clinical practice in upper extremity rehabilitation after stroke.

**Trial Registration:**

ClinicalTrials.gov NCT04446273. Registered on June 23, 2020.

**Supplementary Information:**

The online version contains supplementary material available at 10.1186/s13063-021-05561-6.

## Strengths and limitations of this study

- This study will examine the effects of proximal (i.e., forearm first) and distal (i.e., wrist first) priority robotic therapy combined with impairment-oriented training in stroke rehabilitation.
- This study will investigate the effects of bilateral robotic priming before impairment-oriented training in people with chronic stroke.
- Predictors of treatment outcome will be examined with the chi-square automatic interaction detector method.
- Limitations of this study may include the limited size of the study sample.

## Background

Stroke is a leading cause of death [1] and lost productivity worldwide as measured by disability-adjusted life years [2]. Upper extremity (UE) sensorimotor impairments after stroke, such as weakness, abnormal muscle tone or motor synergies, pain, and hypersensitivity or hyposensitivity, are prevalent and persistent [3] and may further affect activities of daily living [4] and quality of life after stroke [5]. Robot training (RT) is emerging as a novel approach to support therapists in their efforts to provide high-intensity, repetitive, and task-specific interventions. Systematic reviews indicate that RT leads to improvement in UE motor impairment and strength in stroke patients but to less improvement in UE functional use and activities of daily living [6].

### Movement-based robotic priming

Motor priming can be defined as a change in motor performance on the basis of previous stimuli and is an emerging strategy to facilitate motor relearning in neurorehabilitation [7]. Bilateral motor priming involves bimanual, repetitive, and mirror-symmetric movement training before other functional training movements are implemented [7] and may promote normalization of transcallosal inhibition and greater balance in excitability between the hemispheres after stroke for improved functional recovery [8]. Therefore, bilateral symmetrical movements in bilateral motor priming are used as a neuromodulation technique. As an adjuvant intervention, bilateral RT may be combined with different types of functional rehabilitation therapies and may yield differential benefits.

### Bilateral robotic priming before impairment-oriented training

Impairment-oriented training (IOT) is an approach developed to restore body functions after stroke, including remediation of motor impairments, rather than functional performance [9]. Sensorimotor deficits (e.g., single joint movements or quality of motor performance) should be treated specifically according to their individual characteristics. Arm BASIS training and Arm Ability training are two programs of IOT that have been developed for treating different severity levels of UE impairments after stroke. Empirical studies have confirmed the effects of these two programs of IOT on motor recovery, restoration of movement and muscle function, and enhanced quality of motor control [10, 11].

A previous pilot study reported that bilateral robotic priming before IOT was beneficial on UE motor function and muscle power [12]. The protocol [12] included 60 min of RT with two types of movement patterns, consisting of forearm pronation-supination and wrist flexion-extension and abduction-adduction, delivered by the InMotion 3.0 robot [13].

The Rood approach for the treatment of patients with stroke is one of the neurophysiological approaches for the facilitation and inhibition of movement [14]. One basic premise of the Rood approach is the movement of the distal body part in a finely coordinated pattern based on the stability of the proximal body part [15]. However, the Rood approach has been criticized for insufficient empirical evidence. In addition, how the two movement patterns (i.e., forearm pronation-supination and wrist flexion-extension and abduction-adduction) of RT were sequenced in the previous study was not clear [12], and the sample size of the bilateral robotic priming and IOT group was very small (*n* = 10).

This study is therefore designed to extend the previous study [12] by examining the effect of proximal (i.e., forearm first and wrist second) priority robotic priming and impairment-oriented training (PRI) and distal (i.e., wrist first and forearm second) priority robotic priming and impairment-oriented training (DRI) with a larger sample size (i.e., *n* = 20 in each group). We hypothesize that the PRI and DRI groups will both improve significantly on normalized muscle tone, sensorimotor impairment, upper limb function, self-efficacy, and quality of life from preintervention to postintervention and that the improvement will be maintained at the follow-up assessment. In addition, this study will compare the relative effects between PRI and DRI. We hypothesize that the PRI group will achieve better proximal UE motor ability and that the DRI group will achieve better distal UE motor ability.

## Methods and design

### Participants

The inclusion criteria are age between 20 and 75 years, more than 3 months after the onset of a first unilateral ischemic or hemorrhagic stroke, moderate to severe UE motor impairment (total UE score of the Fugl-Meyer Assessment [UEFMA] score between 18 and 56), no severe spasticity in any joints of the affected arm (modified Ashworth Scale score <3 in any of the affected shoulder, elbow, wrist, and fingers), able to follow instructions (Mini-Mental State Examination total score >24), no UE fractures in the past 3 months, and not simultaneously participating in other medication or rehabilitation studies. The exclusion criteria are other neurologic (i.e., epilepsy), neuromuscular, or orthopedic disease, or severe health or physical conditions that might impede participation in this study.

### Design and procedure

The study will use a single-blind randomized pretest and posttest design. Potential participants will be identified by the therapists or research assistants, and the study procedures, risks, and benefits will be explained. All participants will sign the informed consent before enrolling in this study and be allocated to the PRI or DRI groups based on a computer-generated random-sequence table (Table 1). The second author of this study (Yi-chun Li) will generate the allocation sequence, enroll participants, and assign participants to interventions. Allocation concealment is ensured, as the computer-generated random codes are not released. The study will recruit 40 patients with stroke from hospital settings. Initial and outcome assessments will be administered by a well-trained and certified occupational therapist who is blinded to the group assignment, study hypotheses, and intervention of the patients. Trial participants, care providers, outcome assessors, and data analysts will be blinded after assignment to interventions. Unblinding is permissible when knowledge of the actual treatment is absolutely essential for further management of the patient.

**Table 1:**
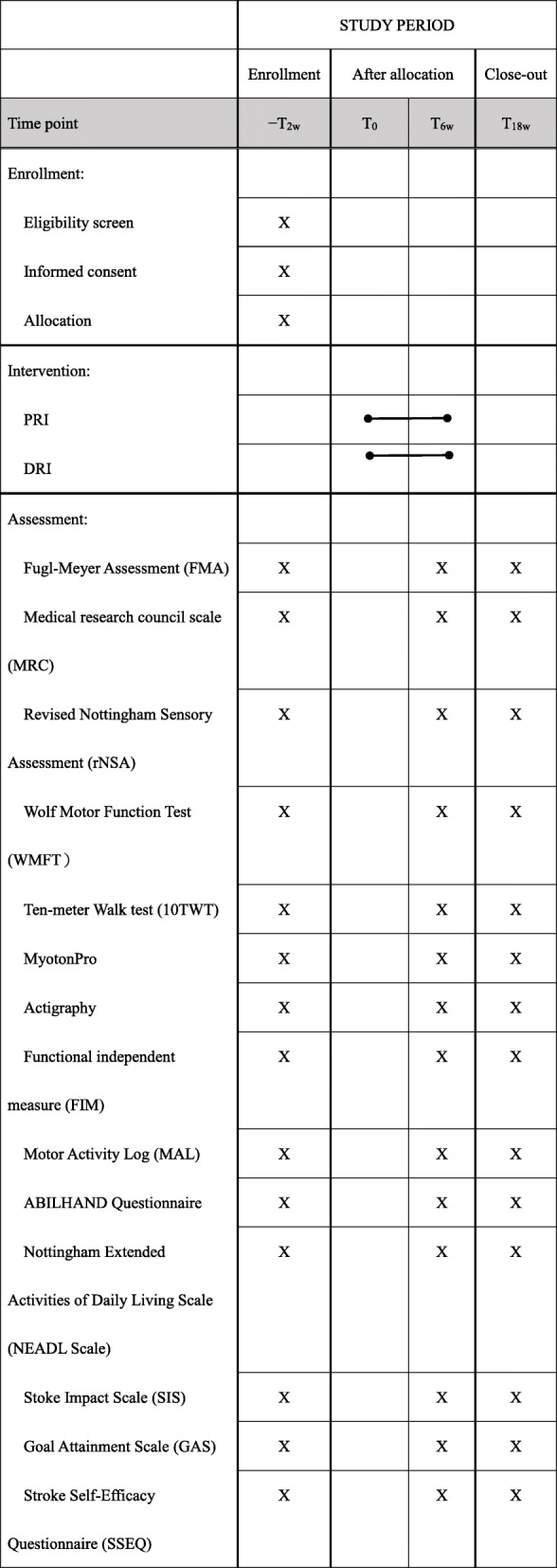
The schedule of enrolment, interventions, and assessments of this study protocol

Outcomes assessment will be administered to participants at baseline, immediately after the intervention, and 3 months after the end of the intervention. The baseline assessment will take place within 1 week before the start of the intervention, and the postintervention assessment will be administered within 1 week after the end of the intervention. Table 1 presents the timing of all study procedures.

### Intervention

The PRI and DRI groups will receive an equal amount of treatment time, comprising 1.5 h per day, 3 days per week, for 6 weeks. All other routine interventions, such as physical therapy or medication, will continue as usual throughout the study period, except for the administration of botulinum toxin.

### PRI group

The PRI protocol provides RT for 45 min and IOT for 45 min. The PRI group will start from the Bi-Manu-Track proximal mode (i.e., forearm RT) and then the distal mode (i.e., wrist RT). In every session of RT, the patient will practice bilateral passive range of motion exercise, in which the unaffected side assists the affected UE, and bilateral active range of motion exercise. RT will be followed by IOT.

The IOT will be delivered according to the participant’s motor ability, namely, the Arm BASIS training (focusing on basic motor control) for severe arm paresis and Arm Ability training (focusing on complex motor control) for mild arm paresis [12]. Participants who score less than 35 in the baseline UE-FMA subscale and cannot achieve a precision grip will be assigned to the Arm BASIS training, and the other participants will receive the Arm Ability training [12].

### DRI group

The DRI protocol is similar to the PRI protocol but starts from the Bi-Manu-Track distal mode (i.e., wrist RT) and then the proximal mode (i.e., forearm RT). The participants will receive 45 min of RT, followed by 45 min of IOT, which is the same as in the PRI group. RT will include passive-passive, active-passive, and active-active range of motion exercise.

### Primary outcome measurements

The **UE-FMA** is used to assess motor impairment of body function, including ranges of motion of the UE joints, and grasping, coordination, and speed of UE movement. The UE-FMA is scored on a 3-point ordinal scale (0 = cannot perform, 1 = perform partially, 2 = perform fully), with a total score between 0 and 55. A higher UE-FMA score means better motor performance and less impairment.

The **Medical Research Council Scale (MRC)** measures muscle power of the affected UE joints. The MRC is scored on a 6-point ordinal scale (0 = plegic, 5 = resisted to maximal strength, full power compared with the unaffected side).

The **Revised Nottingham Sensory Assessment (rNSA)** is used to assess sensory impairment, including pressure, temperature, light touch, kinesthetic sense on the shoulder, elbow, wrist, and hand, and stereognosis. The rNSA is scored on a 3-point ordinal scale (0 = absent, 1 = impaired, 2 = normal), with a total score of 48. The rNSA has acceptable inter-rater reliability.

The **Wolf Motor Function Test (WMFT)** assesses functional motor ability of the UE with 15 functional tasks. The patient is requested to perform tasks as quickly as possible and use the affected side in task performance. Average time (WMFT-Time) and functional ability score (WFMT-FAS) are reported.

### Secondary outcome measurements

The **10-Meter Walk Test (10MWT)** is used to assess walking speed.

The **MyotonPro** is a handheld device that can measure muscle mechanical properties of patients positioned supine.

The **Actigraphy** is a device that is worn on the wrist and may record activity levels of the patient’s UE.

The **Functional Independence Measure (FIM)** is an 18-item measurement tool that assesses a patient’s level of physical, psychological, and social functioning, and disability.

The **Motor Activity Log (MAL)** is a semistructured interview of 30 items and is used to assess the amount of use of the affected UE and the quality of the movement during functional daily activities.

The **ABILHAND Questionnaire** is an inventory of 23 manual activities on which the patient provides his or her self-perceived performing difficulty on a 3-level scale (impossible, difficult, easy) [16].

The **Nottingham Extended Activities of Daily Living Scale (NEADL scale)** is used to measure a patient’s independence in instrumental activities of daily living in the past few weeks across four domains: mobility, kitchen, domestic, and leisure activities.

The **Stoke Impact Scale (SIS) 3.0** is a 59-item questionnaire that measures perceived disability and health-related quality of life after stroke in eight domains: strength, hand function, activities of daily living/instrumental activities of daily living, mobility, communication, emotion, memory/thinking, and participation/role function. A Rasch analysis of the psychometric property of the SIS 3.0 showed good reliability and validity [17].

The **Goal Attainment Scale (GAS)** is an individualized outcome measure for goal setting in an intervention and goal scaling to estimate the extent to which the patient’s goals are met.

The **Stroke Self-Efficacy Questionnaire (SSEQ)** is a 13-item questionnaire that evaluates self-efficacy in functioning after stroke.

### Statistical analyses

The Student *t* test and *χ*^2^ will be used to analyze differences in baseline characteristics and outcome measures between the two groups. Two-way repeated-measures of analysis of variance will be used to determine the intervention effects. The within-subject factor is time (i.e., pretest, posttest, and follow-up assessment), and the between-subject factor is group (i.e., PRI and DRI). A post hoc Tukey test will be used when a group × time interaction or a main effect is observed. An *α* level of .05 is set for all statistical comparisons. Partial eta squared (*η*^2^) will also be estimated to determine the group difference for each outcome measure. Predictors of PRI and DRI outcomes will be estimated with the chi-square automatic interaction detector (CHAID) method. Data analyses will be performed using SPSS Statistics 18.0 software (IBM Corp, Armonk, NY, USA).

### Data management and quality

The documents of this study, including data recording sheets and signed informed consent forms, will be stored in a locked cabinet. The electronic database will be password-protected. Personal information of the participants will be deleted from the electronic database. The principle investigator (Keh-chung Lin) was in charge of data monitoring in this study. Therefore, there was no additional traditional data monitoring committee. The second author of this study (Yi-chun Li) will conduct interim data analyses and the principle investigator (Keh-chung Lin) will make the final decision to terminate the trial.

### Adverse event monitoring and reporting

Pain and fatigue will be monitored during interventions because each session may last 90 min. Patients and the accompanying persons, if any, will also be instructed to report pain, fatigue, and any other adverse events whenever they happen. Adverse events will then be reported to the Ethical Committee for Human Research in accordance with the procedures of the National Taiwan University Hospital and the Feng Yuan Hospital of the Ministry of Health and Welfare, Taichung, Taiwan. Provisions, if any, for ancillary and post-trial care, and for compensation to those who suffer harm from trial participation followed the guidelines of the research ethics committees of the National Taiwan University Hospital and the Feng Yuan Hospital of the Ministry of Health and Welfare in Taiwan.

## Discussion

In recent decades, rehabilitation robots have been rapidly developed and play a critical role in improving UE function after stroke, offering dedicated training that can be coupled with other rehabilitation programs provided by rehabilitation therapists [18]. The effects of combined bilateral robotic priming techniques with task-oriented approaches have been validated in several studies [12, 19]. IOT emphasizes remediation of reduction in UE function after stroke, including deficits in motor control and impaired sensorimotor function of the arm, wrist, and hand [9], but few studies have investigated the combined bilateral robotic priming therapy with IOT. This study is planned to replicate the previous pilot study [12] to examine empirically the immediate and follow-up effects of the combination of bilateral RT with IOT on sensorimotor function of UE in chronic stroke.

For patients with motor control problems, the sequence of relearning of movement from proximal to distal body parts is a topic of debate and needs scientific examination in a systematic way [15]. The proximal joints of the UE, such as the shoulder and scapula, are assumed to be responsible for stability and support of the arm during reaching for targets, and the distal joints (e.g., wrist and hand) are responsible for functional manipulation in activities of daily life. However, distal-emphasized RT (i.e., training on wrist and forearm movements) combined with functional task practice was found to be more effective on muscle strength and quality of movement during functional activities than proximal-emphasized RT (i.e., shoulder and elbow movements) combined with functional task practice [19]. It is possible that distal robotics provide both mechanical stability and repetitive movement practice of the distal joints at the same time, while coordinated distal manipulation of the UE is based on stability from the proximal joints in therapist-facilitated interventions for stroke patients.

In clinical practice, therapists may use techniques targeting several joints of the UE in sequence to promote quality of motor control and functional use of the UE in daily activities. However, no studies to date have investigated the comparative effects of different sequences of training UE joints. Therefore, this study will attempt to extend the previous study [12] by comparing the two combined interventions (i.e., PRI and DRI). Results of this study may provide critical and empirical information about the effects of the training sequence of proximal priority (e.g., forearm first) and distal priority (e.g., wrist first) robotic priming before IOT on motor control and sensorimotor function of UE.

The limited research that is available regarding the effects of bilateral robotic priming combined with IOT provides no information about the responders who are more likely to benefit from this hybrid intervention. Therefore, another goal of this study will be to identify predictors of changes in outcomes after the interventions using the CHAID method. CHAID analysis builds a predictive model to help determine how predicting variables best merge to explain the outcomes in PRI and DRI. CHAID is based on the χ^2^ test to analyze structural linkages between variables and can potentially provide a mechanism for investigating the interaction between demographic and clinical features of patients and outcome measures [20].

Limitations of the design and implementation of this study must also be considered. This is a comparative efficacy study examining two parallel hybrid therapies (i.e., PRI and DRI) and, therefore, does not include a passive control or a conventional rehabilitation group. However, lack of an IOT-only group may limit this study from an estimation of the addictive priming effect of bilateral RT. The sample size of each group (*n* = 20) is small, which reflects the restriction of recruiting large samples of patients with stroke for efficacy studies.

Hemiparesis of the contralateral UE has a significant negative impact on activity participation and social involvement after stroke. There are imperative needs to identify an effective rehabilitation program and characteristics of responders to promote sensorimotor recovery and functional use of the affected UE. The results of this study will provide evidence for the bilateral robotic priming hybrid with IOT on sensorimotor function of UE after stroke. This study will validate the differential effects of sequences in involving joints in UE rehabilitation (i.e., proximal or distal joint first). Implications of these findings will inform the clinical practice of UE rehabilitation in chronic stroke.

### Trial status

The study protocol has not recruited any participants before September 11, 2020. This study protocol is estimated to start participant recruitment on December 1, 2020, and complete recruitment on December 1, 2023. The study protocol was version 4th on the date of 20190110 for ethical review in the National Taiwan University Hospital.

## Supplementary Information

**Additional file 1.**

## Data Availability

Not applicable.

## Abbreviations

PRI: Proximal priority robotic priming and impairment-oriented training
DRI: Distal priority robotic priming and impairment-oriented training
UE: Upper extremity
RT: Robot training
IOT: Impairment-oriented training
FMA: Fugl-Meyer Assessment
MRC: Medical Research Council Scale
rNSA: Revised Nottingham Sensory Assessment
WMFT: Wolf Motor Function Test
WFMT-FAS: WMFT functional ability score
10MWT: Ten-Meter Walk Test
FIM: Functional Independence Measure
MAL: Motor Activity Log
NEADL scale: Nottingham Extended Activities of Daily Living Scale
SIS: Stoke Impact Scale
GAS: Goal Attainment Scale
SSEQ: Stroke Self-Efficacy Questionnaire
CHAID: Chi-Square Automatic Interaction Detector
