# Supplementary material for: Effects of proximal priority and distal priority robotic priming techniques with impairment-oriented training of upper limb functions in patients with chronic stroke: study protocol for a single-blind, randomized controlled trial
Source: Trials. 2021 Sep 8;22:604. doi: 10.1186/s13063-021-05561-6 (PMC8424932; doi:10.1186/s13063-021-05561-6)
Supplement: Supplementary file 1 — Additional file 1. [file 13063_2021_5561_MOESM1_ESM.docx]

**National Taiwan University Hospital**

**Participant Informed and Consent Form for Clinical Trials and Studies**

**Medical record number: ________ Name: ___________ Date of Birth: _________**

Registration number of the research ethics committee in the National Taiwan University Hospital: 202004105RINB

Please read carefully. The principle investigator or the authorized personnel will provide detailed explanation of the study/trial by before you sign this form.

**Study: Effects of proximal priority versus distal priority robotic priming technique with rehabilitation therapy of upper-limb functions in patients with chronic stroke**

Facility: Department of Rehabilitation, National Taiwan University Hospital

Funding Source: National Health Research Institutes, Taiwan

Principle Investigator: Keh-chung Lin

Position: Professor, School of Occupational Therapy, National Taiwan University

Co-Investigator: Yi-hsuan Wu

Position: Occupational Therapist, Department of Rehabilitation, National Taiwan University Hospital

Emergency Contact Person: Keh-chung Lin

Emergency Contact Number: 0918118885 (Please text or leave a voice message when your call is not connected. The researchers will contact you as soon as possible.)

You are invited to participate in this study. This form is to provide information related to this study. The principle investigator or the authorized personnel will explain the details of this study and answer your questions. Before you are satisfied with the answers related to your questions, please do not sign this form. You don’t need to make a decision about participating in this study immediately. Please sign this form to participate in this study after careful consideration. You have to sign this form to participate in this study. This document is a record that you consent to participate in this study. However, you are free to leave the study without any reason even after you agree to participate in this study and sign this form.

1. **Purpose of this study:**

This study plans to examine (1). the effects of proximal priority robotic priming and impairment-oriented training (PRI) and distal priority robotic priming and impairment-oriented training (DRI) on sensorimotor function, motor control, activities of daily living, self-efficacy, and quality of life, (2). at post-intervention and three months after the intervention, and (3). the predictors of intervention.

1. **Background:**

Stroke is one of the major diseases leading to disability in Taiwan. Robotic training (RT) is emerging as a novel approach to support therapists in their efforts to improve UE motor impairment and strength in stroke patients but to less in UE functional use and activities of daily living. Our previous study found RT can be used as warm-up before regular rehabilitation therapy to enhance intervention effects. RT assisted training for upper extremity rehabilitation include proximal (i.e., forearm first and wrist second) priority and distal (i.e., wrist first and forearm second) priority. This study plans to examine the effects of proximal priority RT assisted training and distal priority RT assisted training. The results of our study will provide evidence base for further RT assisted rehabilitation.

1. **Inclusion and exclusion criteria**

The inclusion criteria are age between 20 and 75 years, more than 3 months after the onset of a first unilateral ischemic or hemorrhagic stroke, moderate to severe UE motor impairment (total UE score of the Fugl-Meyer Assessment [UEFMA] score between 18 and 56), no severe spasticity in any joints of the affected arm (modified Ashworth Scale score <3 in any of the affected shoulder, elbow, wrist, and fingers), able to follow instructions (Mini-Mental State Examination total score >24), no UE fractures in the past 3 months, and not simultaneously participating in other medication or rehabilitation studies. The exclusion criteria are other neurologic (i.e., epilepsy), neuromuscular, or orthopedic disease, or severe health or physical conditions that might impede participation in this study.

1. **Procedures of this study**

RT assisted training in this study is carried out with Bi-Manu-Track robot (Reha-Stim Co., Berlin, Germany), a bilateral upper extremity rehabilitation robot for stroke patients. The robot enables two symmetric movements (forearm pronation/supination and wrist flexion/extension) in three treatment modes (passive–passive mode, active–passive mode, and active–active mode). Bi-Manu-Track robot is used as routine rehabilitation in several clinical centers in Germany and Japan. Bi-Manu-Track robot has been approved by the Taiwan Food and Drug Administration and used for training of stroke patients in Departments of Rehabilitation of National Taiwan University and Taipei Veterans General Hospital.

Intervention in this study takes 90 minutes per session, three days a week for 6 weeks. A trained and experienced occupational therapist (not a worker of National Taiwan University) administers intervention in this study. There are two groups of intervention in this study:

**Proximal priority RT assisted training group.** Participants in this group receives RT for 45 minutes and motor training by an occupational therapist for 45 minutes. The RT training will start from the Bi-Manu-Track proximal mode (i.e., forearm RT) and then the distal mode (i.e., wrist RT). After RT, participants will receive individual motor training for 45 minutes.

**Distal priority RT assisted training.** Participants in this group receives RT for 45 minutes and motor training by an occupational therapist for 45 minutes. The RT training will start from the Bi-Manu-Track distal mode (i.e., wrist RT) and then the proximal mode (i.e., forearm RT). After RT, participants will receive individual motor training for 45 minutes.

All participants will sign the informed consent before enrolling in this study and be allocated to the proximal priority RT assisted training and distal priority RT assisted training groups based on a computer-generated random-sequence table. Outcomes assessment will be administered to participants at baseline, immediately after the intervention, and 3 months after the end of the intervention. The baseline and postintervention assessments take about 3 to 6 hours and implement in two sessions. The follow-up assessment takes one and half hours. All the assessments are non-intrusive, and include motor function, sensory function, as well as quality of life. You are asked to do simple movements such as touch your knee, put your hand on your waist, pour water, and grasping a cube, and answer questions related to daily life activities such as “how many times you turn off lights with the affected hand?”, and “How is the strength of your hand this week?”. You can have a 3 to 5 minute break after each test.

1. **Possible adverse event, incidence and management:**

There is no intrusive intervention, injection, nor psychologically stressful event in this study. Therefore, there is no physical or psychological harm to participants. When you feel pain and fatigue during interventions because each session may last 90 minutes, please report pain, fatigue, and any other adverse events to the therapist.

There is no obvious adverse response with the RT (i.e., less than 800 repetitions each mode) and motor training provided in this study according to results of the related studies abroad and our pilot studies. When you have any adverse symptoms such as emotional problems, conscious disturbance, dizziness, or pain, please report to your therapist and stop intervention. You may need to exert muscle power to against the resistance of the robot when perform RT. When you exert muscle power, please breathe regularly. Do not stop breathe nor exert muscle power aggressively, which may induce higher blood pressure or other adverse responses. The therapist will monitor your physical condition, and adjust the treatment accordingly.

Should you experience any adverse response at home, please contact the principle investigator of this study (Keh-chung Lin at mobile phone number 0918118885) or report to the therapist when you receive treatment. Therapists will evaluate if there is any adverse event related to this study. When there is any adverse event related to this study, your participation in this study may be suspended. Provisions, if any, for ancillary and post-trial care, and for compensation to those who suffer harm from trial participation will be implemented following guidelines of the research ethics committees of the National Taiwan University Hospital. When the adverse event is not related to this study, you may drop out of the study if you choose to do so.

1. **Other alternative intervention:**

You are not obligatory to participate in this study. If you do not participate in this study, you will receive routine rehabilitation treatment, which will be arranged by your occupational therapist. You can talk to your attending physiatrist about your treatment program. No matter you choose to participate in this study, your rights to rehabilitation and other health care will not be affected.

Possible benefits of this study

After you participate in the intervention of this study, we anticipate you may progress in areas of motor performance, function in daily life activities, or quality of life. However, the level of progress may be related to severity of stroke.

1. **Contradictions, restrictions or obligations of this study**

During 6-week intervention of this study, please stop the routine occupational therapy. After the end of the 6-week intervention of this study, the routine occupational therapy is permitted. Other intervention (e.g., physical therapy, acupuncture, speech therapy, or other routine rehabilitation) is not restricted. However, Botox^®^ injection to release muscle spasticity of the affected upper extremity is prohibited right after initial assessment and before the end of the follow-up assessment.

1. **Confidentiality and anonymity of participants**

The documents of this study, including data recording sheets and signed informed consent forms, will be stored in a locked cabinet. The electronic database will be password-protected. Personal information of the participants (i.e., name, ID number, address or telephone number) will be deleted from the electronic database. The research ethics committee of National Taiwan University Hospital has periodic independent review of core trial documents and files, and promise to keep your personal information confidential.

1. **Stop or withdraw from this study**

Patients will be participating in this study as volunteers and are free to leave the study without any reason. Stopping or withdrawing from this study will not cause any problem nor influence your rights for future rehabilitation or medical care. For your safety, when you have epileptic attack, serious pain or serious psychiatric symptoms, you have to withdraw from this study.

When there is new and important information which is related to your rights or may influence your participation in this study, we will inform you and have you to decide further participation in this study. Your decision will not cause any problem nor influence your rights for future rehabilitation or medical care. Under such condition, the principle investigator may stop this study.

Upon your withdrawal from this study, we will not collect your data. Your data or personal information will be kept confidential and will not be included in future use.

1. **Compensation and insurance for harm**

There is risk in a trial or study. In order to compensate for possible adverse events for participating in this study, please read carefully the following descriptions.

1. National Taiwan University Hospital take the responsibility for compensation of the adverse events in this study. However, the possible adverse events listed in this consent form are not compensated.
2. National Taiwan University Hospital will provide professional medical care and consultation for adverse events in this study. You do not need to pay for these services.
3. In addition to the above two conditions, this study does not provide any other forms of compensation. If you are not willing to accept the risk, please do not participate this study.
4. You will not lose your legal rights even you sign this consent form.
5. This study does not have any human clinical trial insurance.

If you have adverse events because of this study, the above compensation including reasonable medical cost should comply with the following condition: Your harm is not caused on purpose, and you follow the medical suggestions proposed by the physician in this study.

1. **The storage, use and reuse of specimen and data of the participants**

We will collect data and information with rating scales and questionnaires according to the study proposal and under your authorization during the study period. We will use a code to substitute your name and any other identified personal information after you complete the follow-up assessment or quit from this study. The documents of this study, including data recording sheets and signed informed consent forms, will be stored in a locked cabinet. The electronic database will be password-protected. Personal information of the participants will be deleted from the electronic database. The documents and electronic files will be keep until the end of this study (December 31, 2025). Should your documents and electronic files be used for analysis or statistics in other countries, your legal rights will still be protected under Taiwan’s law. The primary investigator and the research team will do the best to keep your personal information confidential.

1. **Rights of the participants**
2. If you have any questions about the study procedures, concerns about this study, or have possible harm or lost because you participate this study, you can contact the Ethical Committees for Human Research of National Taiwan University Hospital at (02)2312-3456 ext 63155.
3. If we have any significant information which may be related to your health condition or disease and your participation in this study, we will report to you. If you decide to quit this study, your physician will arrange your medical care after this study. If you decide to continue this study, you have to sign a revised version of consent form.
4. If you have any problem or condition during the study period, please do not hesitate to contact professor/occupational therapist Keh-chung Lin in the Department of Occupational Therapy, National Taiwan University Hospital (24-hour contact number: 0918118885).
5. There are two copies of the consent form. The principle investigator or the authorized personnel have given you one copy of the signed informed consent and explained the purpose and procedure of this study. Professor Keh-chung Lin and occupational therapist Yi-hsuan Wu have answered your questions about this study.
6. Funding for this study: No.
7. We will contact you if there is unpredictable but direct influence related to your safety within two years after the end of this study.
8. **Possible commercial interests of this study**

There are no expected patents or any other commercial interests in this study.

1. **Signature**
2. The principle investigator or other authorized personnel have explained the details of this study, including the purpose, procedures and possible risks and benefits.

Signature of the principle investigator or the co-investigator: ________________

Date: __________________

Signature of other research personnel: ____________ Date: _________________

1. I have fully understood this study including the purpose, procedures and possible risks and benefits after detailed explanation and answered questions. I agree to voluntarily participate in this study and possess one copy of the signed consent form.

Signature of the participant: ____________ Date: _________________

Date of birth: ___________________ Telephone: _________________

ID number: _____________________ Gender: □ male □female

Address: ___________________________________________________

Signature of the legal representative: ______________ Date: _________

Relationship with the participant: □spouse □parents □siblings □others

Date of birth: ___________________ Telephone: _________________

ID number: _____________________

Address: ___________________________________________________
